# Risk factor analysis for predicting cervical lymph node metastasis in papillary thyroid carcinoma: a study of 966 patients

**DOI:** 10.1186/s12885-019-5835-6

**Published:** 2019-06-25

**Authors:** Chenxi Liu, Cheng Xiao, Jianjia Chen, Xiangyang Li, Zijian Feng, Qiyuan Gao, Zhen Liu

**Affiliations:** Department of General Surgery, Shengjing Hospital of China Medical University, No.36 Sanhao Street, Shenyang, 100004 China

**Keywords:** Papillary thyroid carcinoma, Lymph node metastasis, Risk factor

## Abstract

**Backgrounds:**

The aim of this study is to investigate the risk factors for the cervical lymph node metastasis in papillary thyroid carcinoma (PTC).

**Methods:**

The clinicopathological data from the 966 PTC patients who underwent thyroid operation between January 2013 and December 2015 in the general surgery department of Shengjing Hospital of China Medical University were collected. The risk factors of predicting cervical lymph node metastasis were analyzed.

**Results:**

Male, age ≤ 45 years old, tumor size> 1.0 cm, extrathyroidal extension (ETE), US features as microcalcification, were independent risk factors for central lymph node metastasis (CLNM) (*P* < 0.05). Only CLNM was independent risk factors for lateral lymph node metastasis (LLNM) (*P* < 0.05). The ROC curve showed that the cutoff value of the number of CLNM for predicting lateral lymph node metastasis was defined as 2.5 (Sensitivity = 0.535, Specificity = 0.722, AUC = 0.669, *P* < 0.05). When the number of CLNM > 3, OR value was significantly higher, suggesting that the risk of LLNM increased significantly. The incidence of LLNM in level III (66.8%) and level IV (67.3%) were significantly higher than level II (42.2%) and level V (21.3%) (*P* < 0.05). The incidence of LLNM and skip metastasis in tumor located in the upper 1/3 of the lobe was the highest (*P* < 0.05).

**Conclusions:**

Prophylactic central lymph node dissection should be performed in patients with risk factors as male, age ≤ 45 years old, tumor size> 1.0 cm, ETE and US features as microcalcification. Lateral lymph node dissection (LLND) should be more actively performed in patients with the number of CLNM> 3. Extent of LLND should include levels II, III, IV and V. Tumor located in the upper 1/3 of the lobe was vulnerable for LLNM and skip metastasis, so lymph node in lateral compartment should be noticed when lymph node status was preoperatively evaluated by imaging examination.

## Background

Thyroid carcinoma is the most common kind of malignant endocrine tumor, accounts for 1% of all human malignant tumors and 33% of the head and neck malignant tumors. Among the thyroid carcinoma, 80–85% are papillary thyroid carcinoma (PTC) [1]. The incidence of cervical lymph node metastasis in PTC can reach to 40–90% [1]. Cervical lymph node metastasis was the main risk factor for a higher recurrence in PTC patient [2, 3]. In general, the lymph node metastasis of PTC occurs in central compartment first**,** then expands to the lateral compartment [4, 5], but it also has the properties of skip metastasis. Therefore, a reasonable and comprehensive initial surgical treatment can decrease the recurrence rate and the reoperation complications. In this study we retrospectively analyzed the clinicopathological data of 966 PTC patients, summarized the features and the risk factors for cervical lymph node metastasis, to help making a reasonable surgical plan and achieve the best treatment effectiveness.

## Methods

### Patients

The study was approved by the Ethical Committee of Shengjing Hospital of China Medical University. We enrolled the patients who underwent the initial thyroid operation in the general surgery department of Shengjing Hospital and were pathologically proved as PTC between January 2013 and December 2015. We excluded the patients with other types of thyroid malignant tumor, without central lymph node dissection (CLND), with the history of thyroid operation, or with incomplete data. There were 966 patients qualified and enrolled in this study.

### Operation approach

Thyroid nodules and cervical lymph nodes were assessed by ultrasound in all patients before surgery. Cervical contrast enhanced computed tomography (CT) and fine needle aspiration (FNA) were not routinely performed during this study and were only used in very few patients. The histology of the frozen sections were performed during the surgical procedures for all the tumors. For the unilateral lobe PTC, lobectomy plus isthmusectomy with ipsilateral CLND was performed; if nodule was detected in the contralateral lobe, total thyroidectomy was performed in our hospital. For the isthmus or bilateral PTC, total thyroidectomy plus bilateral CLND was performed. If lateral lymph node metastasis (LLNM) is evident on preoperative imaging exam or proved by fine needle aspiration cytology, a functional lateral lymph node dissection (LLND) would be performed.

### Clinicopathological properties

The following clinicopathological properties were studied to analyze the risk factors for the CLNM: gender, age, preoperative TSH level, unilateral/bilateral location of tumors in the lobes, the number and size of tumor, with or without extrathyroidal extension (ETE), distant metastasis, or Hashimoto’s thyroiditis (HT), or US features including microcalcification, hypoechoic solid nodules, irregular shape, infiltrative margins, and intra-nodular vascularity. In addition to the above variables, the status of central lymph node was also considered as a clinicopathological property for analyzing the risk factors for the LLNM. Solitary was defined as one tumor in the thyroid, multifocality was defined as two or more tumors in the thyroid. Tumor size was defined as the maximal diameter in solitary case, and was defined as the maximal diameter of the largest tumor in the multifocality cases. ETE was defined as invading strap muscle, larynx, trachea, esophagus, recurrent laryngeal nerve, prevertebral fascia, encasing carotid artery or mediastinal vessels. TSH level was measured within one month before surgery in our hospital, and the normal range was 0.3–4.8uIU/mL. The HT patients were diagnosed with any one of the following criteria: positive for anti-thyroid peroxidase (TPO) antibody; positive for antithyroglobulin antibody; pathologic confirmation of HT [6].

### Statistics analysis

Software SPSS 17.0 was used for statistics analysis. Univariate analysis was performed with univariate logistic models, and variables with statistical significance in univariate analysis were further included in the multivariate logistic models. The multivariate analysis was performed with binary logistic regression analysis to assess independent risk factors for CLNM and LLNM. ROC curve was used to determine the critical value of the number of CLNM for predicting LLNM. Differences were assessed with the chi-square test for categorical variables. Measurement data such as TSH level, the average age and the average tumor size was presented as mean ± standard deviation. Statistical significance was considered when *P* < 0.05.

## Results

### Patients’ characteristics

Among the 966 cases, including 194 men and 772 women, the average age was 45 ± 12 years old (ranging from 9 to 80 years old). The average tumor size was 1.34 ± 1.02 cm, ranging from 0.1 to 6 cm. The range of TSH value was 0.001–14.68 uIU/mL. 767 cases were unilateral PTC, among which 679 cases were solitary, 88 cases were multifocality. There were 199 bilateral PTC cases. All patients underwent CLND, CLNM was found in 367(38.0%) cases. 420 patients underwent the functional LLND, 211 cases had LLNM, including 155 cases with LLNM and CLNM simultaneously and 56 cases with skip metastasis. Among the solitary cases, LLNM occurred in 130 cases, of which 36 cases had skip metastasis. Surgical complications included: 138 cases of transient hypoparathyroidism(14.3%), 12 cases of permanent hypoparathyroidism(1.2%), 9 cases of unilateral vocal cord paralysis(0.9%), 2 cases of bilateral vocal cord paralysis(0.2%), 5 cases of postoperative hemorrhage(0.5%), and 8 cases of chylous leakage(0.8%). Postoperative radioiodine therapy was performed on 252 patients. All patients were followed up after surgery until February 2018. The median follow-up time was 40 months (range, 25–61 months). During the follow-up period, none of patients died. 15 cases (1.6%) experienced recurrence, including 12 cases with lymph node recurrence and 3 cases with thyroid recurrence. The patients with disease recurrence received an additional surgery.

### Risk factors for CLNM

Univariate analysis showed that CLNM was significantly associated with age, male, tumor size, multifocality, bilateral location of tumors in the lobes, ETE and US features as intra-nodular vascularity or microcalcification (*P* < 0.05). However, distant metastasis, HT, the TSH value and US features as hypoechoic solid nodules, irregular shape or infiltrative margins were not significantly associated with CLNM (*P* > 0.05) (Tables 1 and 2). The multivariate analysis showed age ≤ 45 years old, male, tumor size> 1.0 cm, ETE and US features as microcalcification were the independent risk factors for CLNM (*P* < 0.05) (Table 3).

**Table 1:** Demographics and clinical characteristics of patients undergoing central lymph node dissection

Clinicopathological properties			Central lymph node metastasis			
		Total	No		Yes	
Subjects, n		966	599	62.0%	367	38.0%
Gender	Female	772	496	64.2%	276	35.8%
	Male	194	103	53.1%	91	46.9%
Age (years)	≤35	229	97	42.4%	132	57.6%
	35–45	248	146	58.9%	102	41.1%
	45–55	273	193	70.7%	80	29.3%
	> 55	216	163	75.5%	53	24.5%
Tumor size(cm)	≤0.5	178	145	81.5%	33	18.5%
	0.5–1.0	363	255	70.2%	108	29.8%
	1.0–2.0	275	140	50.9%	135	49.1%
	> 2.0	150	59	39.3%	91	60.7%
Ultrasound feature						
Microcalcification	No	706	459	65.0%	247	35.0%
	Yes	260	140	53.8%	120	46.2%
Hypoechoic solid nodules	No	183	112	61.2%	71	38.8%
	Yes	783	487	62.2%	296	37.8%
Irregular shape	No	631	401	63.5%	230	36.5%
	Yes	335	198	59.1%	137	40.9%
Infiltrative margins	No	252	158	62.7%	94	37.3%
	Yes	714	441	61.8%	273	38.2%
Intra-nodular vascularity	No	328	228	69.5%	100	30.5%
	Yes	638	371	58.2%	267	41.8%
Multifocality	No	679	439	64.7%	240	35.3%
	Yes	287	160	55.7%	127	44.3%
Bilateral	No	767	491	64.0%	276	36.0%
	Yes	199	108	54.3%	91	45.7%
Extrathyroidal extension	No	759	500	65.9%	259	34.1%
	Yes	207	99	47.8%	108	52.2%
Distant metastasis	No	961	598	62.2%	363	37.8%
	Yes	5	1	20.0%	4	80.0%
Hashimoto’s thyroiditis	No	604	384	63.6%	220	36.4%
	Yes	362	215	59.4%	147	40.6%
TSH value			2.10 ± 1.65		2.13 ± 1.49

**Table 2:** Univariate analysis of risk factors for central lymph node metastasis

		OR	95%CI		*P* value
			Lower	Upper	
Gender	(male vs. female)	1.59	1.16	2.18	0.004
Age (years)					< 0.001^a^
	≤35	4.19	2.79	6.28	< 0.001
	35–45	2.15	1.44	3.51	< 0.001
	45–55	1.28	0.85	1.91	0.240
	> 55	1			
Tumor size(cm)					< 0.001^a^
	≤0.5	1			
	0.5–1.0	1.86	1.20	2.89	0.006
	1.0–2.0	4.24	2.71	6.62	< 0.001
	> 2.0	6.78	4.11	11.18	< 0.001
Ultrasound feature					
Microcalcification	(yes vs. no)	1.59	1.19	2.13	0.002
Hypoechoic solid nodules	(yes vs. no)	0.96	0.69	1.33	0.803
Irregular shape	(yes vs. no)	1.21	0.92	1.58	0.176
Infiltrative margins	(yes vs. no)	1.04	0.77	1.40	0.793
Intra-nodular vascularity	(yes vs. no)	1.64	1.24	2.18	0.001
Multifocality	(yes vs. no)	1.45	1.10	1.92	0.009
Bilateral	(yes vs. no)	1.50	1.09	2.06	0.012
Extrathyroidal extension	(yes vs. no)	2.11	1.54	2.88	< 0.001
Distant metastasis	(yes vs. no)	6.59	0.73	59.19	0.092
Hashimoto’s thyroiditis	(yes vs. no)	1.19	0.91	1.56	0.195
TSH value		1.01	0.93	1.10	0.799

^a^ means the global *p*-values

**Table 3:** Multivariate analysis of risk factors for central lymph node metastasis

		OR	95%CI		*P* value
			Lower	Upper	
Gender	(male vs. female)	1.48	1.04	2.11	0.028
Age (years)					< 0.001^a^
	≤35	4.83	3.11	7.48	< 0.001
	35–45	2.62	1.70	4.04	< 0.001
	45–55	1.50	0.97	2.32	0.066
	> 55	1			
Tumor size(cm)					< 0.001^a^
	≤0.5	1			
	0.5–1.0	1.55	0.98	2.46	0.060
	1.0–2.0	3.22	2.00	5.18	< 0.001
	> 2.0	4.85	2.81	8.35	< 0.001
Microcalcification	(yes vs. no)	1.43	1.04	1.97	0.029
Intra-nodular vascularity	(yes vs. no)	1.30	0.95	1.78	0.100
Multifocality	(yes vs. no)	0.96	0.59	1.59	0.886
Bilateral	(yes vs. no)	1.39	0.80	2.41	0.242
Extrathyroidal extension	(yes vs. no)	1.68	1.18	2.39	0.004

^a^ means the global *p*-values

### Risk factors for LLNM

**Fig. 1:**
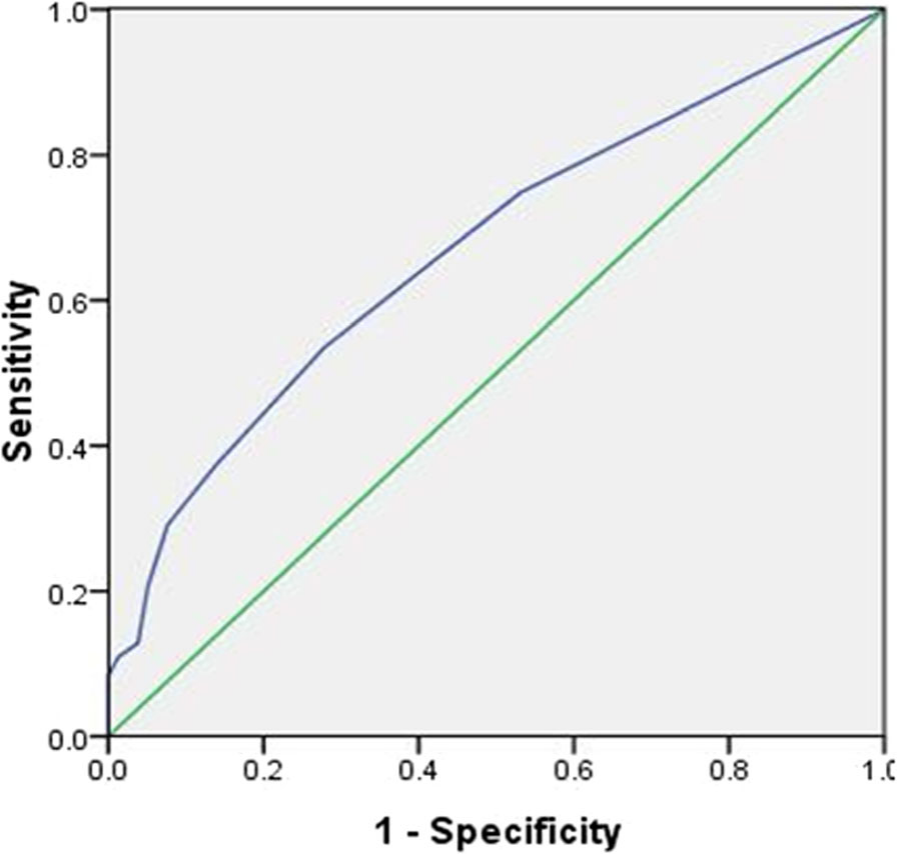
Receiver operating characteristic curve analysis of the number of central lymph node metastasis for predicting lateral lymph node metastasis. The results of ROC showed that the cutoff value of the number of central lymph node metastasis was 2.5, which was the optimal point to distinguish between PTC with and without lateral lymph node metastasis. At this value, the sensitivity was 53.5% and the specificity was 72.2%; AUC was 0.669, and the 95% CI was 0.599–0.739

In 420 patients who underwent LLND, we analyzed the risk factors for LLNM. The univariate analysis showed that LLNM was statistically significant associated with age, tumor size, ETE and CLNM (*P* < 0.05). LLNM was not significantly related with gender, US features, tumor number, unilateral or bilateral location of tumors, distant metastasis, HT, or the TSH value (*P* > 0.05) (Tables 4, 5). We found that there was a significant difference in incidence of LLNM between PTC with and without CLNM. In order to further study the relationship between the number of CLNM and the incidence of LLNM, we made ROC curve to determine the critical value of the number of CLNM for predicting LLNM in 234 cases with CLNM. As shown in Fig. 1, the cutoff value of the number of CLNM was 2.5 (Sensitivity = 0.535, Specificity = 0.722, AUC = 0.669, *P* < 0.05). Therefore we grouped the number of CLNM as: none, 1–3 and > 3 in all models for LLNM. In the multivariate analysis, we found that only CLNM was the risk factor for LLNM, and with the increase of the number of CLNM, the OR value increased, the OR value of the number of CLNM> 3(OR = 9.27) was more than 3 times that of the number of CLNM equal to 1–3(OR = 2.96), which suggested that the risk of LLNM increased significantly with the increase of the number of CLNM (*P <* 0.05) (Table 6).

**Table 4:** Demographics and clinical characteristics of patients undergoing lateral lymph node dissection

Clinicopathological properties			Lateral lymph node metastasis			
		Total	No		Yes	
Subjects, n		420	209	49.8%	211	50.2%
Gender	Female	329	172	52.3%	157	47.7%
	Male	91	37	40.7%	54	59.3%
Age (years)	≤35	121	48	39.7%	73	60.3%
	35–45	104	52	50.0%	52	50.0%
	45–55	108	56	51.9%	52	48.1%
	> 55	87	53	60.9%	34	39.1%
Tumor size(cm)	≤0.5	35	24	68.6%	11	31.4%
	0.5–1.0	137	82	59.9%	55	40.1%
	1.0–2.0	147	69	46.9%	78	53.1%
	> 2.0	101	34	33.7%	67	66.3%
Ultrasound feature						
Microcalcification	No	277	144	52.0%	133	48.0%
	Yes	143	65	45.5%	78	54.5%
Hypoechoic solid nodules	No	73	33	45.2%	40	54.8%
	Yes	347	176	50.7%	171	49.3%
Irregular shape	No	259	131	50.6%	128	49.4%
	Yes	161	78	48.4%	83	51.6%
Infiltrative margins	No	95	48	50.5%	47	49.5%
	Yes	325	161	49.5%	164	50.5%
Intra-nodular vascularity	No	114	57	50.0%	57	50.0%
	Yes	306	152	49.7%	154	50.3%
Multifocality	No	272	142	52.2%	130	47.8%
	Yes	148	67	45.3%	81	54.7%
Bilateral	No	315	163	51.7%	152	48.3%
	Yes	105	46	43.8%	59	56.2%
Extrathyroidal extension	No	286	153	53.5%	133	46.5%
	Yes	134	56	41.8%	78	58.2%
Distant metastasis	No	416	207	49.8%	209	50.2%
	Yes	4	2	50.0%	2	50.0%
Hashimoto’s thyroiditis	No	251	130	51.8%	121	48.2%
	Yes	169	79	46.7%	90	53.3%
The number of CLNM	0	186	130	69.9%	56	30.1%
	1–3 > 3	165 69	68 11	41.2% 15.9%	97 58	58.8% 84.1%
TSH value			2.20 ± 1.66		2.16 ± 1.44

**Table 5:** Univariate analysis of risk factors for lateral lymph node metastasis

		OR	95%CI		*P* value
			Lower	Upper	
Gender	(male vs. female)	1.60	1.00	2.56	0.051
Age (years)					0.025^a^
	≤35	2.37	1.35	4.17	0.003
	35–45	1.56	0.88	2.78	0.132
	45–55	1.45	0.82	2.57	0.206
	> 55	1			
Tumor size(cm)					< 0.001^a^
	≤0.5	1			
	0.5–1.0	1.46	0.66	3.23	0.346
	1.0–2.0	2.47	1.13	5.40	0.024
	> 2.0	4.30	1.89	9.81	0.001
Ultrasound feature					
Microcalcification	(yes vs. no)	1.30	0.87	1.95	0.205
Hypoechoic solid nodules	(yes vs. no)	0.80	0.48	1.33	0.392
Irregular shape	(yes vs. no)	1.09	0.74	1.61	0.671
Infiltrative margins	(yes vs. no)	1.04	0.66	1.64	0.865
Intra-nodular vascularity	(yes vs. no)	1.01	0.66	1.56	0.953
Multifocality	(yes vs. no)	1.32	0.88	1.97	0.175
Bilateral	(yes vs. no)	1.38	0.88	2.15	0.160
Extrathyroidal extension	(yes vs. no)	1.60	1.06	2.43	0.026
Distant metastasis	(yes vs. no)	0.99	1.38	7.10	0.992
Hashimoto’s thyroiditis	(yes vs. no)	1.22	0.83	1.81	0.311
The number of CLNM					< 0.001^a^
	0	1			
	1–3	3.31	2.13	5.15	< 0.001
	> 3	12.24	5.98	25.06	< 0.001
TSH value		0.98	0.86	1.11	0.711

^a^means the global *p*-values; *CLNM* central lymph node metastasis

**Table 6:** Multivariate analysis of risk factors for lateral lymph node metastasis

		OR	95%CI		*P* value
			Lower	Upper	
Age (years)					0.447^a^
	≤35	1.64	0.87	3.09	0.125
	35–45	1.26	0.66	2.40	0.478
	45–55	1.46	0.78	2.75	0.236
	> 55	1			
Tumor size(cm)					0.060^a^
	≤0.5	1			
	0.5–1.0 1.0–2.0	1.57 1.94	0.67 0.84	3.63 4.51	0.297 0.121
	> 2.0	3.03	1.24	7.44	0.015
Extrathyroidal extension	(yes vs. no)	1.26	0.78	2.02	0.350
The number of CLNM					< 0.001^a^
	0	1			
	1–3	2.96	1.87	4.70	< 0.001
	> 3	9.27	4.38	19.63	< 0.001

^a^ means the global *p*-values; *CLNM* central lymph node metastasis

### The features of the LLNM

In 211 cases with LLNM confirmed by pathology, the metastasis rates of level III (66.8%) and IV (67.3%) were significantly higher than that of level II (42.2%) and V (21.3%) (*P* < 0.05), but the difference between level III and IV was not statistically significant (*P* > 0.05). So, LLNM most likely occurs in level III and IV (Table 7). Moreover, lymph metastasis often involved multiple levels in the lateral compartment, multiple level lymph node metastasis was found in 129 (61.1%) cases (4 levels in 18 cases, 3 levels in 40 cases and 2 levels in 71 cases). Single level lymph node metastasis in lateral compartment occurred in 82 (38.9%) cases (12 cases in level II, 31 cases in level III, 35 cases in level IV and 4 cases in level V).

**Table 7:** The incidence of lateral lymph node metastasis in different sites of lateral cervical compartment

The sites of lateral compartment	Lateral lymph node metastasis		Positive rate	*χ* ^*2*^	*P* value
	No	yes			
				123. 60	< 0.001
Level II	122	89	42.2%		< 0.001^a^, < 0.001^b^, 0.918^c^
Level III	70	141	66.8%		< 0.001^d^, < 0.001^e^, < 0.001^f^
Level IV	69	142	67.3%		
Level V	166	45	21.3%		

^a^Level II vs. Level III, ^b^Level II vs. Level IV, ^c^Level III vs.Level IV, ^d^Level II vs. Level V,

^e^ Level III vs. Level V, ^f^Level IV vs. Level V

To determine whether the location of the tumor is related to the occurrence of LLNM, we analyzed LLNM in 272 patients with solitary tumor who underwent LLND. We found that tumor located in the upper 1/3 of the lobe had the highest LLNM incidence (60%) (*P* < 0.05) (Table 8). To determine whether the location of the tumor was related to the level in lateral compartment of lymph node metastasis, 130 cases of solitary tumor with lateral lymph node metastasis were analyzed. We found that the location of the tumor was not related to the level in lateral compartment of lymph node metastasis (*P* > 0.05) (Table 9).

**Table 8:** Analysis about the tumor location and lateral lymph node metastasis

Tumor location	lateral lymph node metastasis		Positive rate	*χ* ^*2*^	*P* value
	No	Yes			
				11.423	0.022
Upper 1/3 of the lobe	38	57	60.0%		
Middle 1/3 of the lobe	34	20	37.0%		
Lower 1/3 of the lobe	47	29	38.2%		
Isthmus	14	13	48.1%		
Whole	9	11	5.5%

**Table 9:** Relation between tumor location and the sites of lateral lymph node metastasis

Tumor location	The site of lateral lymph node metastasis				*χ* ^*2*^	*P* value
	Level II	Level III	Level IV	Level V		
					9.621	0.649
Upper 1/3 of the lobe (*n* = 57 cases)	34	38	36	15		
Middle 1/3 of the lobe (*n* = 20 cases)	5	10	18	5		
Lower 1/3 of the lobe (*n* = 29 cases)	8	16	19	5		
Isthmus (*n* = 13 cases)	3	8	10	2		
Whole (*n* = 11 cases)	4	7	10	2		

We also found that in 130 cases of solitary tumor with LLNM, 36 cases did not have CLNM, that is the so-called “skip metastasis”, which have negative ipsilateral CLNM and positive ipsilateral LLNM. Further analysis found that tumors located in upper 1/3 of the lobe had the highest skip metastasis incidence (*P* < 0.05), however, the location of the tumor was not related to the level in lateral compartment of skip metastasis (Tables 10, 11).

**Table 10:** Analysis between the tumor location and skip metastasis

Tumor location	Skip metastasis		Metastasis rate	*χ* ^*2*^	*P* value
	No	Yes			
				11.124	0.021
Upper 1/3 of the lobe	35	22	38.6%		
Middle 1/3 of the lobe	18	2	10.0%		
Lower 1/3 of the lobe	21	8	27.6%		
Isthmus	9	4	30.8%		
Whole	11	0	0%

**Table 11:** Relation between tumor location and the sites of skip metastasis

Tumor location	The site of skip metastasis				*χ* ^*2*^	*P* value
	Level II	Level III	Level IV	Level V		
					6.689	0.674
Upper 1/3 of the lobe (*n* = 22 cases)	15	10	11	3		
Middle 1/3 of the lobe (n = 2 cases)	1	1	2	1		
Lower 1/3 of the lobe (*n* = 8 cases)	1	2	4	2		
Isthmus (*n* = 4 cases)	1	1	3	1		

## Discussion

Although PTC is the most common pathological type of thyroid carcinoma with a 10-year survival exceeding 90% [7], previous studies found that cervical lymph node metastasis was common for PTCs and 40–90% of all PTCs could occur cervical lymph node metastasis [1, 8–10]. It is widely accepted that cervical lymph node metastasis is a major cause of the local recurrence of PTC and it may also influence patients’ survival [11–13]. Study showed reoperation for PTC recurrence was relatively difficult and might significantly increase the surgical complications which would affect patient’s quality of life [14]. So, the treatment of cervical lymph nodes during initial operation is very important for the prognosis of patients. At present, there is still controversy about whether prophylactic CLND and the extent of therapeutic LLND. The main reasons for the controversy are as follows: first, CLND has potential higher incidence of complications and uncertainty of improved outcome; second, there is no evidence for what extent of LLND is the most appropriate for the management of LLNM. Therefore, for guiding cervical lymph node dissection, it is of great significance to explore the properties and risk factors of cervical lymph node metastasis in PTC patients.

### Risk factors for CLNM

Same with some of the previous results, in our study male was a risk factor for CLNM [15–19], which suggested that CLNM had a gender tendency. In males patients, physical examination and imaging evaluation of cervical lymph node status should be emphasized preoperatively. Whether age is related to CLNM, the current findings are not consistent. Liu et al [20] found that CLNM were not correlated with age, while other studies reported that age < 45 years old was a risk factor for CLNM [17–19]. In this study, we found that younger age was associated with a higher odds ratio of CLNM, and age ≤ 45 years old was independent risk factor for CLNM, which suggested that CLNM should be noticed in the younger patients.

In this study, we found that microcalcification on the US image was a risk factor for CLNM. Microcalcification is a calcium salt deposition due to hyperplasis of blood vessels and fibrous, reflecting rapid growth of cancer cells. Therefore, if microcalcification is found in the nodules, the lymph node status in central region should be assessed more carefully [21].

Multifocality, bilateral tumor and ETE were already included in previous studies as clinicopathological characteristics, there were studies showing that multifocality, bilateral tumor and ETE were the risk factors for CLNM [17, 22–24], but our study showed that ETE was the risk factor for CLNM, which may be due to that once the tumor cells invade the thyroid capsule, it is easy to transfer to the surrounding lymph nodes along the rich lymphatic tissue around the capsule.

Tumor size was always considered as an important predictive factor for cervical lymph node metastasis in PTC, but the cutoffs were different. Ahn et al [18] showed that tumor size≥1 cm was the risk factor for CLNM, while Yan et al [17] considered that tumor size≥0.25 cm. Furthermore, some studies reported that cervical lymph node metastasis was positively related to the primary tumor’s size, as the size of the tumor increased, the incidence of cervical lymph node metastasis increased [17, 19, 25]. Our study divided the tumor size into 4 groups based on the AJCC staging system and the definition of PTMC. The multivariate analysis showed that the larger tumor was associated with an increased odds of CLNM, and tumor size> 1.0 cm was the independent risk factor for CLNM. So we considered that the tumor size> 1.0 cm was threshold for CLNM.

### Features and risk factors for LLNM

There were many studies about the features and the risk factors for LLNM, but few of them had comprehensive clinicopathological properties, and the results of those studies were controversy. Zhang et al [16] reported that ETE, bilateral tumor and CLNM were risk factors for LLNM. Niel et al [26] considered that tumor located in upper pole, CLNM, and tumor size> 1.5 cm were the risk factors for LLNM, but Lin et al [19] considered that CLNM wasn’t risk factor for LLNM. In this study, we found that only CLNM was the independent risk factor for LLNM; and the OR value increased with the increase of the number of CLNM, the OR value of the number of CLNM> 3(OR = 9.27) was more than 3 times that of the number of CLNM equal to 1–3 (OR = 2.96) (*P <* 0.05), which suggested that CLNM number > 3 was much more prone to LLNM than CLNM number equal to 1–3. So when the number of CLNM> 3, the LLND should be more actively performed.

To date, the extent of therapeutic lateral neck dissection for PTC remains unclear. Several authors have reported that PTC metastasis is generally present at level II to V in lateral compartment, and lateral neck dissection including levels II to V is necessary for complete clearance of lateral neck metastasis [27–29]. But some authors raise objections to routine level V dissection for PTC patients with lateral compartment lymph node metastasis [30, 31]. In our study, lymph node metastasis in the lateral compartment occurred mostly in level III and IV, and multiple levels involvement was common. Our data consistent with the idea that extent of lateral central neck dissection should include levels II, III, IV and V.

In most of the previous studies, the solitary tumor location was divided into 4 groups as the upper 1/3, middle 1/3, lower 1/3 and isthmus, the relationship between tumor location and LLNM was examined [16, 32, 33]. Zhang et al [16] demonstrated that the primary tumor in the upper 1/3 of the lobe had a lower risk for CLNM and a higher risk for LLNM. Qubain et al [33] showed that tumor in the upper 1/3 of the lobe was more vulnerable to transfer to lymph node in the upper cervical region, and the tumor in the isthmus and the lower 1/3 of the lobe was more likely to transfer to lymph node in the lower cervical region. Furthermore, it has been found that the skip metastasis of LLNM often happens in tumor located in the upper 1/3 of the lobe [16, 34]. Lei et al [34] reported that 39 patients with lymph node skip metastasis in their study had a much higher rate of level II but a lower rate of Level III, IV and V lymph node involvement. In our study, the solitary tumor location was divided into 5 groups as the upper1/3, middle1/3, lower1/3, isthmus and whole which means tumor occupying the whole lobe. We found that among the 272 solitary tumor patients who underwent LLND, tumor located in the upper 1/3 of the lobe had the highest incidence of LLNM. Furthermore, by analyzing 130 solitary tumor cases with LLNM, we found skip metastasis was more vulnerable to occur in tumor located in upper 1/3 of the lobe, which might be due to the upper 1/3 lymphatic vessels of the lobe draining into the deep lateral lymph nodes, which are in common carotid artery bifurcation along the superior thyroid artery and vein. However, we didn’t find that the location of the tumor was related to the level in lateral compartment of lymph node metastasis and skip metastasis in our study, which may be due to the small sample of solitary tumor with LLNM and skip metastasis, further investigation should be done by expanding the sample.

In this study, the positive rate of metastasis was not high in patients who underwent lateral neck dissection, only 50.3%. The possible reason for this is that we assessed lymph node metastasis primarily by ultrasound, contrast enhanced CT and FNA were only used in very few patients during the study. The sensitivity and specificity of ultrasound in assessing cervical lymph node metastasis is not high, and the results of ultrasound depends on the operator’s diagnostic experience to a large extent, which leads to a low positive rate of lateral neck dissection performed according to ultrasound. To solve this problem, we now routinely perform cervical contrast enhanced CT, ultrasound-suspected metastatic lymph nodes are further assessed by FNA, which can reduce unnecessary LLND. The combination of the US/CT/FNA/intraoperative biopsy could achieve a very high sensitivity and specificity in assessing cervical lymph node metastasis.

## Conclusions

In summary, we considered that prophylactic CLND should be performed in patients with risk factors as male, age ≤ 45 years old, tumor size> 1 cm, and ETE and US features as microcalcification. LLND should be more actively performed in patients with the number of CLNM> 3. Extent of LLND should include levels II, III, IV and V. Lymph nodes status should be preoperatively assessed by US, CT and FNA. For tumor located in the upper 1/3 of the lobe, LLNM and skip metastasis was likely occurred, so lateral lymph node should be noticed when lymph node status was preoperatively evaluated by imaging examination.

## Data Availability

All data analyzed during this study are available from the corresponding author on reasonable request.

## Abbreviations

AJCC: American Joint Committee on Cancer
CLND: Central lymph node dissection
CLNM: Central lymph node metastasis
ETE: Extrathyroidal extension
HT: Hashimoto’s thyroiditis
LLND: Lateral lymph node dissection
LLNM: Lateral lymph node metastasis
PTC: Papillary thyroid carcinoma
TNM: Tumor node metastasis
TSH: Thyroid stimulating hormone
